# Digital financial services for health in support of universal health coverage: qualitative programmatic case studies from Kenya and Rwanda

**DOI:** 10.1186/s12913-023-09893-8

**Published:** 2023-09-28

**Authors:** David Randolph Wilson, Sherri Haas, Sicco Van Gelder, Regis Hitimana

**Affiliations:** 1 Management Sciences for Health, Arlington, VA USA; 2 Credence Management Solutions, LLC, Supporting U.S. Agency for International Development, Washington, D.C USA; 3 PharmAccess Foundation, Amsterdam, The Netherlands; 4 Rwanda Social Security Board, Kigali, Rwanda

**Keywords:** Digital financial services, Digital health, Universal health coverage, Rwanda, Kenya, mHealth, Health insurance

## Abstract

**Background:**

This document describes two qualitative programmatic case studies documenting experiences implementing digital financial services (DFS) for health with a focus on expanding access to universal health coverage (UHC). The CBHI 3MS system in Rwanda and the i-PUSH and Medical Credit Fund programs in Kenya were selected because they represent innovative use of digital financing technologies to support UHC programs at scale.

**Methods:**

These studies were conducted from April-August 2021 as part of a broader digital financial services landscape assessment that used a mixed methods process evaluation to answer three questions: 1) what was the experience implementing the program, 2) how was it perceived to influence health systems performance, and 3) what was the client/beneficiary experience? Qualitative interviews involved a range of engaged stakeholders, including implementers, developers, and clients/users from the examined programs in both countries. Secondary data were used to describe key program trends.

**Results:**

Respondents agreed that DFS contributed to health system performance by making systems more responsive, enabling programs to implement changes to digital services based on new laws or client-proposed features, and improving access to quality data for better management and improved quality of services. Key informants and secondary data confirmed that both implementations likely contributed to increasing health insurance coverage; however, other changes in market dynamics were also likely to influence these changes. Program managers and some beneficiaries praised the utility of digital functions, compared to paper-based systems, and noted their effect on individual savings behavior to contribute to household resilience.

**Discussion/Conclusions:**

Several implementation considerations emerged as facilitators or barriers to successful implementation of DFS for health, including the importance of multisectoral investments in general ICT infrastructure, the value of leveraging existing community resources (CHWs and mobile money agents) to boost enrollment and help overcome the digital divide, and the significance of developing trust across government and private sector organizations.

The studies led to the development of five main recommendations for the design and implementation of health programs incorporating DFS.

## Background

About 100 million people are pushed into extreme poverty (living on $1.90 or less a day) each year because of out-of-pocket (OOP) spending on health.^1^ This extreme poverty caused by high OOP spending is especially felt by women as they typically have more restricted access to financial and productive assets than men, and they shoulder a greater burden of using unpaid leave time to care for sick family members. Gender inequality is high in many countries facing high or extreme poverty rates, and women in low- and middle-income countries (LMICs) are less likely than men to own mobile phones and to access internet-based mobile services.^2^

Digital financial services (DFS) for health can contribute to attaining the Sustainable Development Goal 3.8 of achieving universal health coverage (UHC).^3^ DFS applications include digital health insurance; health savings accounts; credit, transfers, remittances, and loans for health purposes; vouchers for health care; payments for health care/insurance by beneficiaries; and bulk purchases/payments across the health system, including payments to health workers.

Financial protection is achieved when direct payments made to obtain health services do not expose people to financial hardship and do not threaten living standards. A key to protecting people is to ensure prepayment (savings) and pooling of resources (insurance) for health, rather than paying for services out-of-pocket at the time of use. As found in a recent systematic review^2^, advances in digital technology have made it more efficient and affordable to reach people with these key services. For example, digital loans can smooth health and non-health expenditures, digitization of health insurance processes results in operational and cost efficiencies and DFS have the potential to improve service quality.

While there is evidence that DFS for health can improve programs designed to increase access to UHC,^4^ there are still many challenges impeding the operationalization and uptake of DFS for health. This research was conducted to examine how specific programs addressed these challenges and the role DFS may have played in advancing financial protection, accessing health services, and supporting improved health system performance. The case studies documented through this research are:

- Rwanda: Community‐Based Health Insurance (CBHI) program
- Kenya: M-TIBA-based i-PUSH program and Medical Credit Fund (MCF) loans including Cash Advance (CA) and Mobile Asset Financing (MAF)

**Fig. 1:**
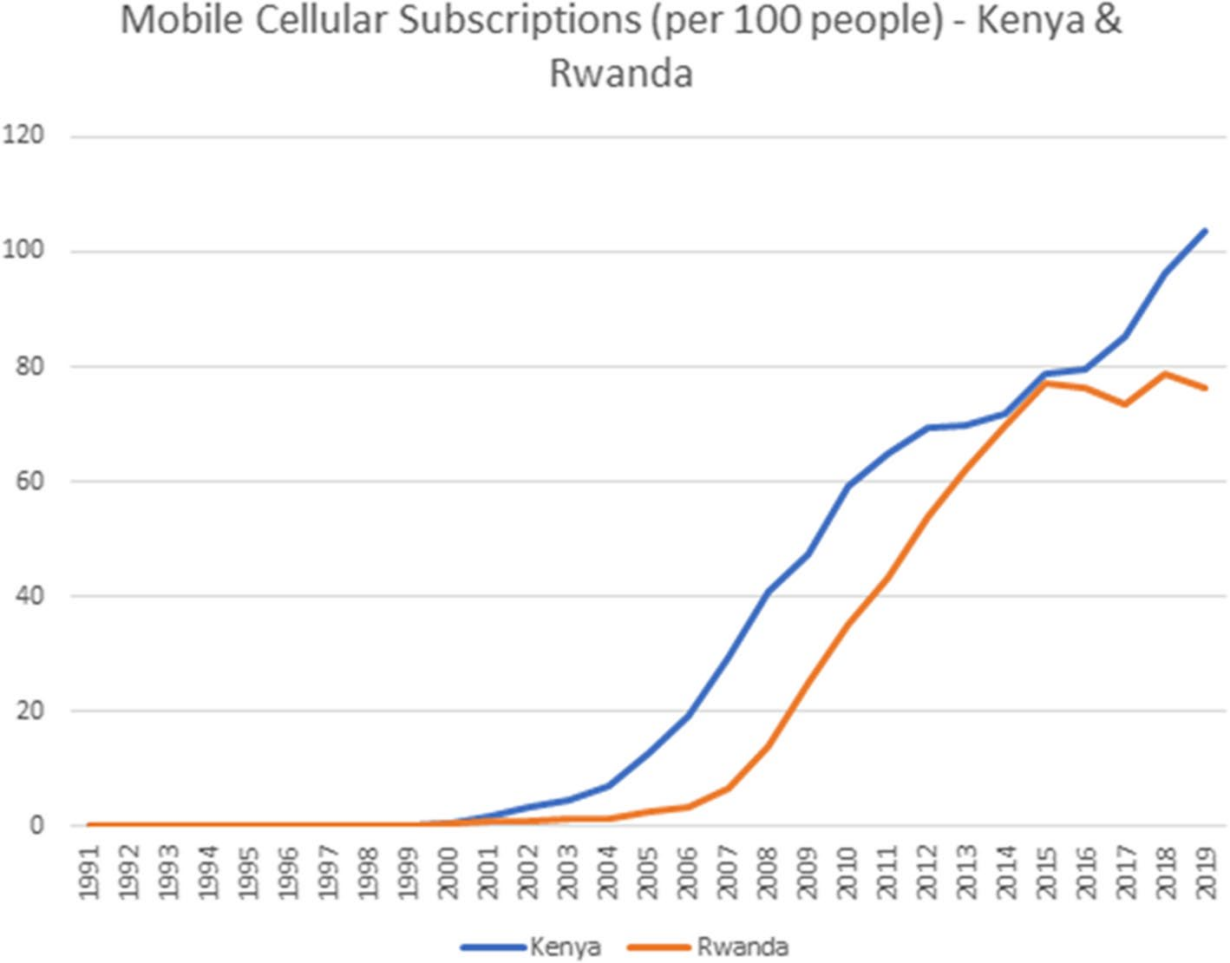
(**a**) Mobile Cellular Subscriptions (per 100 people)—Kenya & Rwanda. (**b**) The blue curve indicates the trend in mobile cellular phone subscriptions per 100 people in Kenya (**c**) The red curve indicates the trend in mobile cellular phone subscriptions per 100 people in Rwanda. (**d**) Both countries saw a dramatic increase in mobile phone penetration from around 2002 until 2014. At that time Rwandan subscriptions plateaued, while the Kenyan subscriptions continued to increase to the point where there was slightly more than 1 phone subscription per capita. This mobile phone penetration has laid a strong foundation for enabling access to digital financial services for health

Both of these countries have seen rapid increases in mobile phone penetration over the past decade^5^ (see Fig. 1) and have in place national health insurance schemes designed to benefit the majority of the population that falls outside of the formal employment sector.

The goal of this research was to help inform more widespread integration of DFS in health by answering the following questions from the perspectives of the broad range of actors engaged in system implementation (program implementers, health service providers and national authorities) and use (insured beneficiaries and health service providers)^6^:

1. What was the experience in implementing the DFS program?
  a Facilitators and barriers to successful implementation
  b Program adaptations
  c Pandemic-related changes
2. How was the program perceived to influence health system performance?
3. What has been the client/beneficiary experience of the program with regard to:
  a Financial protection
  b Service demand/utilization

This detailed case study approach enables us to better understand the rationale for specific DFS interventions, highlight implementation issues encountered and draw out recommendations to inform future DFS initiatives to support advancement toward UHC.

## Methods

This manuscript relates to one component of a broader landscape assessment of digital financial services in support of universal health coverage produced for Digital Square that used a mixed methods approach (key informant, program client/beneficiary interviews and secondary analysis of quantitative data on beneficiary demographics and service utilization). This study focuses only on the qualitative component that used key informant and client/beneficiary interviews to examine the key implementation considerations of the programs. While other countries have designed and implemented digital financial tools to support health insurance (e.g., Ghana National Health Insurance),^7^ these programs were selected because they represent innovative use of digital financing technologies to support UHC programs at scale. The Rwandan “Mutuelle Membership Management System” (3MS) is government-led and implemented nationally, while the i-PUSH and CA/MAF programs in Kenya are privately led and target specific underserved populations—women of reproductive age from low-income communities and their children.

The digital solutions examined were quite different. In Rwanda, 3MS was a custom-developed software focused initially on enrolling and validating beneficiaries, then in a second phase, it created interfaces to mobile payment services. In Kenya, the i-PUSH program combined several different digital interventions from the start: a digital system for enrolling beneficiaries and managing payments to insurers and providers, in addition to two digital loan programs managed by the Medical Credit Fund (MCF) which utilize the same underlying technology of CarePay’s M-TIBA platform^8^: Cash Advance (CA) for operational costs and Mobile Asset Financing (MAF) to manage a loans program for cash-strapped service providers. The programs in each country interfaced with the respective government systems for participant identification.

Data collection methods included qualitative key informant interviews (KIIs) and client/beneficiary interviews. The study sought to create a 360° view of DFS programs by engaging the broad range of stakeholders involved in the programs. A stratified purposeful sampling methodology was used to select participants to conduct semi-structured KIIs. Participants were selected based on several criteria as described in Table 1.

**Table 1:** Key informants selected for each research question

Question	Key Informants
1. Implementation Experience	Implementers: insurance scheme managers, software developers, health facility managers and NGO program managers
2. DFS influence on health systems performance	Implementers as stated above and program beneficiaries
3. Client/beneficiary experience	Program beneficiaries (insurance enrollees and health facility managers accessing digital loans)

For full transparency, some of the study investigators were involved in the implementation of some of these DFS programs: Management Sciences for Health (MSH) supported – through USAID/Rwanda support under the Rwanda Integrated Health Systems Strengthening Project (IHSSP) and the Rwanda Health System Strengthening Activity (RHSS) – the Ministry of Health and Rwanda Social Security Board (RSSB) in the design and implementation of Rwanda’s 3MS system and PharmAccess staff lead implementation of the i-PUSH and MCF loans programs. This provided a high level of access to insights about program implementation and secondary data. All field data collection was done by non-affiliated researchers (Table 2).

**Table 2:** Number of interviews conducted by country

Country	Implementers	Beneficiaries
Kenya	7	26
Rwanda	9	18

Interview guides were created for each research question. Experienced data collectors were hired to conduct the field interviews in Kinyarwanda and Kiswahili and transcribe them into English. The data collectors pre-tested the translated interview guides and made minor changes to the translations before completing the process. Field interviews were recorded and transcribed, as were about half of the above site key informant interviews (copious notes were taken for the other above site interviews). The recorded interviews were transcribed using transcription software (otter.ai) and some light editing was required to correct a few undiscernible quotes based on interview notes.

A data-charting approach^9^ was used to extract the interview data, and analysts followed a 3-level coding approach to group responses according to themes. The selection of themes was done both deductively and inductively. The initial set of themes was built around the qualitative study questions and responses to each question were first classified under these headings. These were further subdivided into sub-themes before beginning the data charting analysis.

The data charting process involved developing an Excel analysis grid with each question and response coded in rows and six sets of thematic codes in columns (four to seven text strings each related to demand, enrollment, barriers, outcomes, sustainability and other). Each of the responses was reviewed by a data analyst and the content was manually linked to the appropriate thematic code. A third level of themes emerged out of some of the interviews—particularly when the interviewers probed for “Other outcomes” related to health system performance—and the analysts revisited the data charts a third time to see if any of these new themes had been missed.

Finally, Excel’s table filtering tools were used to select responses related to each theme and extract them into a synthesized table that grouped responses to each theme by DFS program (Rwanda -CBHI, Kenya—i-PUSH and Kenya—MAF/MCF). This synthesized table was used by the authors to easily compare findings across programs and formed the basis for the narratives in the results section.

As described in the study protocols approved by ethics committees in each country, survey respondents were advised of the voluntary nature of their contributions and provided written (in the case of face-to-face interviews) or verbal consent (for virtual interviews) before participating in the study.

Key informant interviews were conducted from January through April 2021 as follows:

Secondary data was also used to compare enrollment trends from CBHI in Rwanda and PharmAccess in Kenya. In Kenya this information allowed us to also estimate the proportion of households that enrolled in year 1 of i-PUSH when premiums were fully subsidized who continued into year 2, when beneficiaries had to cover a substantial portion of the cost themselves (the ‘transition rates’).

## Results

This section describes key results as they relate to the research questions.

### I. What was the experience implementing the DFS program?

#### Facilitators and Barriers to Successful Implementation

Respondents from both case studies highlighted a variety of common factors that contributed to successful DFS solution implementation. These included technology factors such as: existing mobile/internet network infrastructure and mobile money network operators; strong software development teams; high penetration of mobile phone use across all target clients; the existence of web-enabled systems facilitating automatic, real-time verification using national ID numbers to ensure the correctness of data and secure electronic financial transactions; a population open to using mobile money for financial transactions—both at the individual and business level (healthcare providers).

People factors were also highlighted. The programs were implemented through initiatives designed to bring together multidisciplinary teams of stakeholders (technology, policy/government administration, health, and finance) from public, private and NGO sectors and building trust between stakeholders (a crucial but sometimes slow process) to enable data sharing and interoperability between independently managed systems.

Both programs benefited from the existence of trusted and functional community service systems. In Kenya, the programs were able to leverage the trusted community health volunteers and workers networks that had established relationships in the community—especially in rural Kakamega County where mothers feared going to hospitals but trusted local health workers from their own community. In Rwanda, the program was able to build upon the network of district- and community-based representatives from *Irembo*^10^ (a public private partnership to enable digital payment for government services) and mobile money agents in remote areas, helping less digitally literate citizens complete their transactions and overcome the digital divide.

The health financing policy environment was also a key enabler in DFS implementation. In both case studies, the DFS schemes were built on top of pre-established functioning ecosystems of health financing (e.g., CBHI in Rwanda; National Hospital Insurance Fund [NHIF] in Kenya). In Rwanda, respondents noted that the government’s policy and vision of digital transformation for government financial services, with the motto “zero paper, zero trips,” drove the change with strong political support. In Kenya, the DFS innovations were driven more by nongovernmental organizations (PharmAccess and AMREF) with social enterprise missions who effectively engaged public and private sector stakeholders.

Both programs also encountered barriers and challenges along the way that required mitigation. Key among these on the technology side were: difficulty negotiating data sharing agreements to enable interoperability between systems managed by different stakeholders (government – national identification (ID) and household/birth registration; insurers—NHIF and CBHI enrollment systems; mobile operators—gateways for financial transactions) and issues related to software platforms themselves (some systems lacked APIs, computer interfaces needed to be updated as systems changed, and some insurance schemes were digitized, which prevented program managers from identifying gaps or overlaps in coverage). This was compounded by the lack of a comprehensive data privacy regulatory framework in both countries.

**Fig. 2:**
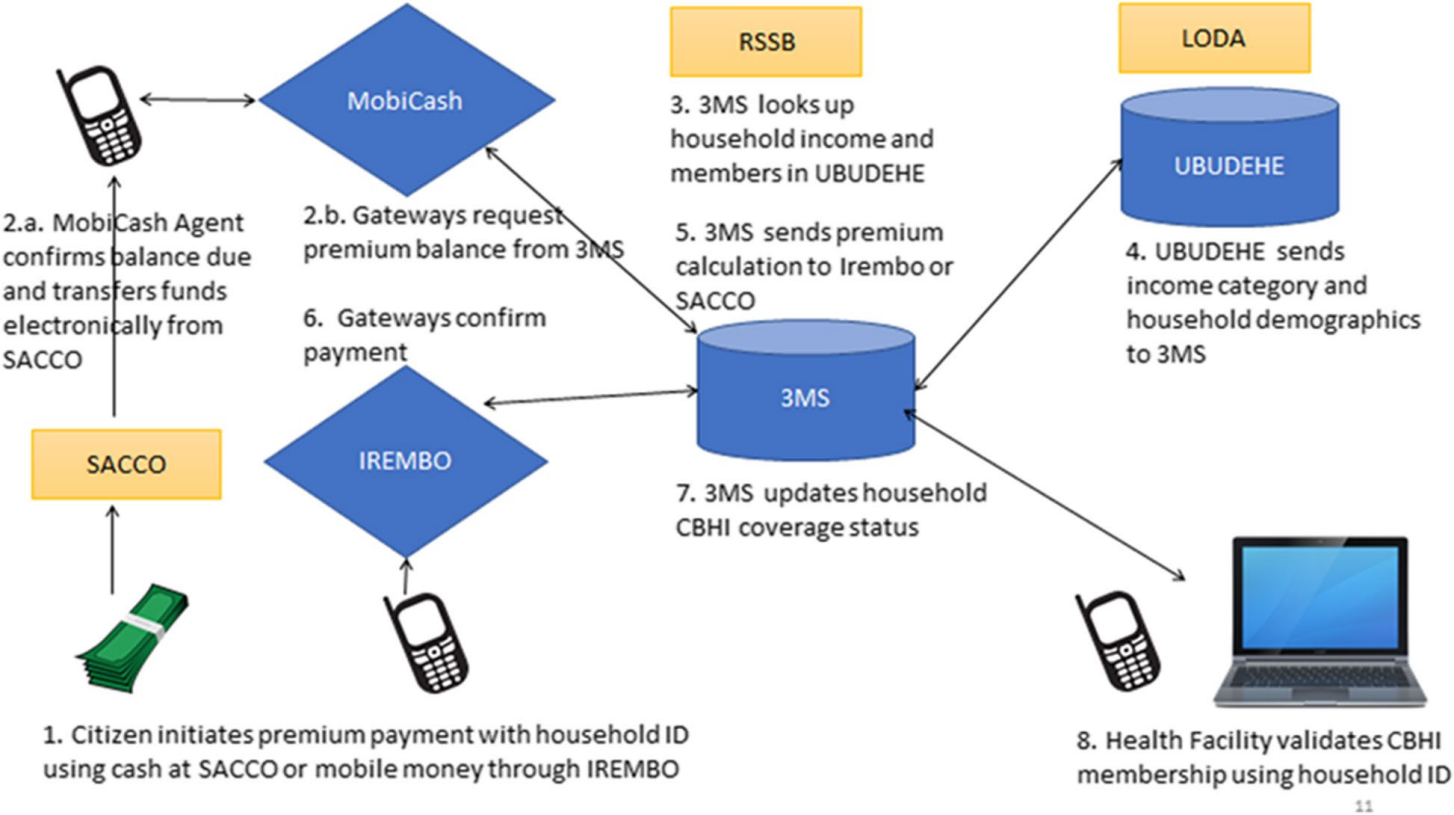
(**a**) Interoperability schema and key functions supported by Rwanda 3MS. (**b**) This simplified flow chart describes how CBHI premium payments can be initiated by citizens using either cash payments through SACCO (Saving and Credit Cooperative Society) or directly using their mobile money accounts on personal mobile phones. In either case, a mobile phone is used to process the transactions. These include checking the household membership and income category through the 3MS database and sending back the total premium cost and receiving the payment. Once the premium is paid, the system automatically updates the household members’ CBHI coverage status. Health facilities can then validate CBHI membership status for patients using a mobile phone or computer. Through a separate process, household income classification data are synchronized regularly with the UBUDEHE household income classification database maintained by LODA (Local Administrative Entities Development Agency)

When the Rwanda 3MS development started, there was no interoperable payment gateway available for government programs so considerable effort went into creating custom connections between different digital platforms (Fig. 2). Further, some health facilities had spotty internet connectivity which led RSSB staff to attempt an offline system that failed due to challenges synchronizing with the centralized databases.

On the user/beneficiary side, clients and community level agents with low levels of digital literacy were challenged in using some of the DFS services that required familiarity with smartphones. Some of the Rwandan CBHI staff noted that training was insufficient – as the cascaded orientation sessions often focused more on policy changes to the CBHI scheme than on practical exercises using the software. Users in both countries faced financial hardship, exacerbated by COVID-19, and had difficulty making household financial decisions with their limited resources. One i-PUSH program respondent demonstrated the competing priorities they faced: *“Do I buy food? Do I pay premium? Do I pay school fees?”.*

Just as traditional banks have been cautious about lending to private healthcare small and medium enterprises (SMEs) with no credit history or obvious collateral for loans, some health facility leaders were concerned about participating in the mobile lending programs. *“Health facility leadership were careful, they were wary, they were even fearful that you want to tap into the M-PESA events, virtual accounts” [when they agreed to share tier financial data].—MCF program respondent.*

The following table summarizes key barriers and facilitators (Table 3):

**Table 3:** Summary of key implementation facilitators and barriers

Facilitators*	Barriers^a^
**• Existing mobile/internet network infrastructure and mobile money network operators**	**• **Old infrastructure not up to peak demand; spotty internet in some remote areas (CBHI 3MS)
**• Strong software development teams**	**• **Lack of electronic payment gateways and APIs to reliably connect systems managed by different actors (CBHI 3MS)
**• High penetration of mobile phone use and providers and beneficiaries open to or using mobile money for financial transactions**	**• **Poor quality smart phones produce inadequate images of required certificates for registration (i-PUSH)
**• Existence of web-enabled systems enabling automatic, real-time verification using national ID numbers**	**• **Government health facilities could not apply for mobile credit funds (MCF)
**• **Existing mobile money agent network to extend reach of DFS services (CBHI 3MS)	**Low-income households had too many competing demands on their limited income and no experience with savings**
**• **Strong community of Kenyan software developers available to manage and improve the M-TIBA platform (i-PUSH and MCF)	
**• **Government’s digital vision: “Zero paper, zero trips” catalyzed change (CBHI 3MS)	Inadequate onboarding of CBHI and facility staff using 3MS (CBHI 3MS)
**• Large network of CHWs to assist with household-level enrollment and bridge the digital literacy divide**	**• Absence of comprehensive policies or health worker capacity building on cybersecurity and management of protected health information (PHI) impeded data sharing between stakeholders**

^a^Facilitators and barriers in bold were identified across both cases. Where a factor was only identified in one case, it is specified in parentheses

### Program Adaptations

Barriers identified during program implementation needed to be mitigated in order for the programs to succeed. Program managers from both case studies highlighted examples of how online platforms built to support DFS enabled the programs to be more agile and quick to implement policy changes (e.g., changing insurance coverage wait times, increasing loan limits), tweak the system to make incremental changes and improvements to the user interface, fix bugs, and introduce new features requested by clients. This helped to respond to the challenge of telecommunications companies that were also innovating constantly, so the DFS platforms could evolve and adapt quickly to their innovations.

### Pandemic-Related Changes

The COVID-19 pandemic resulted in challenges and opportunities for the DFS programs in both countries. In general, when it came to savings and paying insurance premiums, people had more difficulty paying because of loss of revenue—and job losses hit hardest in the poorer communities that were served by the insurance schemes.

On the other hand, COVID-19's distancing mandates, suggested precautions, and heightened health awareness helped to accelerate uptake of digital health platforms. Both the Rwandan and Kenyan governments encouraged more electronic payments to reduce in-person interactions. In Rwanda, citizens were motivated to enroll in CBHI because they perceived a greater likelihood of getting sick.

Rwandan CBHI program managers felt that the DFS system contributed to resilience during COVID-19, as payments could be made from home during lockdown, unlike through traditional channels that handled cash payments such as banks that reduced work hours and were less geographically accessible. Citizens’ ability to access these services remotely also reduced potential exposure to COVID-19 virus.

In Kenya, the i-PUSH enrollment work faced challenges throughout the pandemic. As some of this required in-person interface with households, the program had to cease activity when surges resulted in lockdowns. Further, requirements around social distancing, wearing proper personal protective equipment, and limiting large group gatherings forced the need for additional trainings with smaller groups at higher cost. Loan agents also faced challenges initially, as they were forced to adapt their marketing efforts to health facilities remotely; however, they adapted by doing more virtual phone calls. Businesses, like hospitals, turned more to MCF’s loans to cover losses in revenue and to purchase specialized equipment required to treat COVID-19 patients (e.g., ventilators).

### II. How was the program perceived to influence health systems performance?

Respondents from both case studies perceived that the DFS for health programs contributed to improved health system performance, including aspects of data quality and use, and improved quality of care, responsiveness, and efficiency. The DFS initiatives also supported national eGovernment initiatives to move from manual to automated management for greater efficiency, transparency, and control.

#### Data Quality and Use

An unanticipated theme that emerged from the KIIs was that both systems promoted use of data by clients and providers and contributed to a heightened awareness of the importance of data quality. The system developers incorporated features to enhance data quality—such as linking to national ID databases to validate ID numbers and retrieve accurate identification data and implementing artificial intelligence image recognition controls in mobile apps to ensure that appropriate documents are being uploaded during the registration process. Effective multi-sectoral coordination is needed to establish relationships between different ministries who manage health services, financial technology (FINTECH) and civil registration in order to set up data sharing agreements. Respondents perceived that the availability of information through mobile phone interfaces enabled citizens and health facility managers to be more engaged in the process, including for checking coverage status, making payments, knowing exact payment requirements, and confirming household members covered in real-time.

In different ways, all programs contributed to enabling different stakeholders to use data more effectively for evidence-based decision-making. For example, 3MS provided more accurate, disaggregated, and timely data on premium revenues and membership campaign coverage than earlier aggregate reporting tools.

#### Efficiency and Financial Considerations

The DFS for health programs supported national eGovernment initiatives to move from manual to automated management with the potential for greater efficiency and transparency. Some respondents observed that digital systems made it possible to quickly implement changes to their services based on new laws or client-proposed features, making systems more responsive and adaptable. They also noted that citizens have more trust in financial transactions placed through the private sector DFS ecosystem. There was no intermediary (e.g., insurance agent counting on commissions from enrollments) and a perception by enrollees that there was less chance of fraud. Similarly, third party insurance payments that are common in Rwanda’s CBHI – such as relatives in urban areas paying the CBHI premiums for unemployed family members in rural areas, or small businesses paying the premiums for families of their day laborers—were sure to be used for intended purpose as they went directly to RSSB.

The programs also contributed to potential cost efficiencies. In Rwanda, the introduction of 3MS reduced the number of cash financial transactions managed at the facility level and digitized the labor-intensive work of managing paper household record systems thereby requiring fewer staff. While new posts were created at district CBHI sections and at the national level to manage a heavier workload at higher levels—and there were costs associated with orienting staff to the new technologies—the net result was reported to be cost savings. The system also enabled new types of facilities with no CBHI staff (e.g., health posts and telemedicine providers) to check eligibility with a simple SMS message before providing services. Digital membership verification was much more efficient and protected the providers from the risk of not being reimbursed.

The CBHI scheme was also more efficient. Under the old system, Savings and Credit Cooperatives (SACCOs) were collecting money daily and then transferred funds at the end of the month. In fact, there were often substantial delays in this process that worked to the benefit of the SACCOs – who maintained these funds to give loans to their members and earn additional revenues – in addition to the commission they charged on each premium received. Now money is transferred immediately to RSSB. One CBHI manager estimated that *“at the end of the year the amount of interest generated by getting the funds into the CBHI pool quickly is bigger than the commissions that are paid to SACCOs and mobile money agents for the transactions.”*

Facilities praised the simplicity of using the MCF systems, especially digital cash advance. The ease and speed of the process helped to ensure that SMEs could not only access financial resources, but could do so when they needed it, making the service much more responsive and improving their ability to provide services to clients.

#### Quality of care

Some of the efficiencies enabled by digitizing financial and membership management services were perceived to improve quality of care. In both case studies, respondents shared that they were motivated to seek care earlier because they no longer risked paying high out-of-pocket expenses for care. A CBHI program manager in Rwanda noted, we* “don’t expect that we will be able to show measurable change in quality of care [through this qualitative study], but clients receive better service—less time waiting in queues, and immediate triage for life saving care”.*

The mobile phone applications that support M-TIBA and 3MS enabled citizens to be more engaged with the program. Using a simple mobile feature phone, they could send an SMS to the 3MS system to check the status of their coverage, make payments, know how much to pay, and confirm household member coverage in real-time. Clients of CBHI expressed that service had improved, as prior to the use of digital payments there was a one-month processing delay between paying for the CBHI cover and accessing services. When paying digitally, the client gains access immediately.

In Kenya, the digital financing services were accompanied by significant effort to make sure facilities were working toward quality improvement. The SafeCare program’s digital quality checklists played a role in this, by enabling regular quality assessments in hospitals and linking them to resources through CA/MAF to help make improvements.

Patient registration and eligibility checking also goes more smoothly because verification is done in real time by phone or computer. In Rwanda, this responsibility was transferred from dedicated staff hired by CBHI in each facility to frontline health workers who checked this during triage without patients having to pass through a separate insurance queue as in the past. Many respondents indicated that the introduction of the digital systems has made paying for and accessing health services quicker. *“When you get to the hospital, you don’t need to queue; you just use your mobile phone to activate your account, bring out your name and you are quickly attended to”. – i-PUSH participant.*

Similarly, i-PUSH beneficiaries could choose from a wider range of health providers and could choose to go where more services were offered or providers were perceived to offer better quality of service. This led to increased income in preferred facilities that in turn enabled providers to increase the scope of services they provided, creating a virtuous cycle. *“We've seen facilities who had earlier no laboratory services, but they were able to generate more income and [were] able to increase the services by opening labs in their facility”. – i-PUSH NHIF program manager.*

Interviews demonstrated that DFS programs have other unintended benefits for the participating healthcare providers. For instance, the record-keeping and data reporting required within the programs encouraged additional rigor in accounting, bookkeeping, and other administrative management practices. The programs inspired a culture of quality program and health facility management in a way that was not planned but was welcomed by all. *“It became so easy to inculcate that culture of quality management in terms of healthcare because of this program. Many hospitals were [came] up with quality improvement teams and … there was a lot of training for both medical and non-medical staff through this program. This of course improved their efficiency and productivity [leading to improved] quality of services rendered to the patients.” – i-PUSH NHIF program manager.*

### III. What was the client/beneficiary experience of the program?

**Fig. 3:**
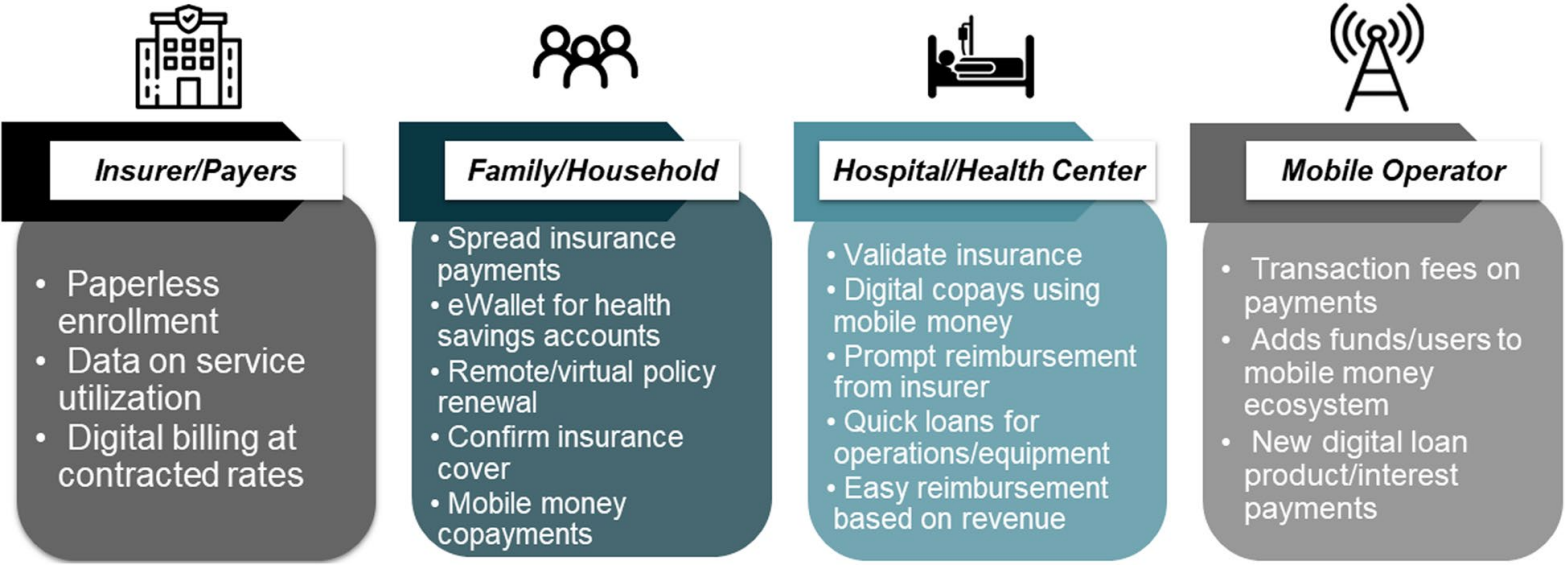
(**a**) Perceived benefits of DFS programs by stakeholder group. (**b**) This graphic highlights key stakeholders in the DFS program ecosystem and the benefits that they perceived from using the digital financial services described in this case study. Source: Study authors, based on interviews and literature review

The study respondents identified a wide range of benefits that they perceived to accrue from the programs documented by these case studies. The following chart highlights some of the key benefits across different actors engaged in DFS for health (Fig. 3):

The following sections provide more detailed evidence of some of these benefits as they relate to key sub-components of the beneficiary experience.

#### Financial protection

Key informants perceived that both programs (3MS and i-PUSH) contributed to increasing insurance coverage (though the DFS tools were only one of many changes in market dynamics that influenced this change). Many interviewees praised the ease of use of all functions of the system—enrollment, payment, and accessing services. Clients could easily make payments from home, helping them to make payment on time, retain coverage, and remain able to access services. “*When we used to pay for example NHIF, we were to go to Kiambu so as to pay but for now that we are paying *via* the phone you can pay at any time even at night.” – i-PUSH participant.*

The i-PUSH program’s digital enrollment tools enabled registration of many beneficiaries in a short period. *“[i-PUSH] managed to enroll more than 35,000 women and their households who had never been on insurance. Giving them a platform, where based on the frequency of their incomes they can put in money in small bits and save for their healthcare.” – PharmAccess program manager.*This supports the finding in a previous systematic review that “mobile money accounts help people smooth health and non-health expenditures when faced with a health shock.”^3^

DFS systems in both countries relied on socioeconomic mapping to identify poor households to target for subsidies and enroll for access to health services. In Rwanda, linking 3MS to the *Ubudehe*^11^ income classification database enabled RSSB to identify those households that fell into the indigent category and enroll them automatically in CBHI with premiums fully paid by the government – while those who could afford to pay were charged on a sliding scale. In fact, the introduction of the progressive premium structure – rather than the simple standard premium for all in the original CBHI scheme – proved to be impossible to implement without the digital platform to lookup a household’s income category in order to determine how much they should pay.

Similarly, i-PUSH’s socioeconomic mapping gave the *“government information about who they should subsidize, especially the poorest of the poor, and encouraged other partners/NGOs to contribute by subsidizing NHIF enrollments for households that couldn't afford the whole enrollment fee.” – i-PUSH program staff.* Because many clients never had any health coverage before, i-PUSH represented their first opportunity to feel secure in accessing health services without risking a bill they would not be able to pay. Beyond practical financial considerations, respondents described an improved state of mind and confidence. For some clients “*the shift from the one-time large payment of NHIF to gradual saving made paying for health coverage easier.*” *i-PUSH client.*

These examples highlight how DFS programs can contribute to increased and more equitable access to health services.

On the provider side, facilities participating in MCF’s Cash Advance and Mobile Asset Financing programs indicated that access to credit improved the ability of facilities to weather dips in funding and consistently pay expenses, maintaining a more stable supply of medications and ensuring health workers are paid. Some respondents indicated that the loans were especially critical in helping them remain solvent through the worst points of the COVID-19 pandemic.

A major advantage to loans through CA and MAF was predictability. Clients reported a greater predictability about whether or not they would receive a loan and how much they qualified for, which allowed them to plan more effectively. Facilities praised the repayment directly from the till, with many reporting that the gradual repayment linked to revenues eased the financial management burden and avoided difficult situations at the end of the month. *“I can say the fact that the money is being deducted from the till, is what is making us feel like we don’t have that weight of repayment.”—MCF client facility.*

#### Service demand/utilization

**Fig. 4:**
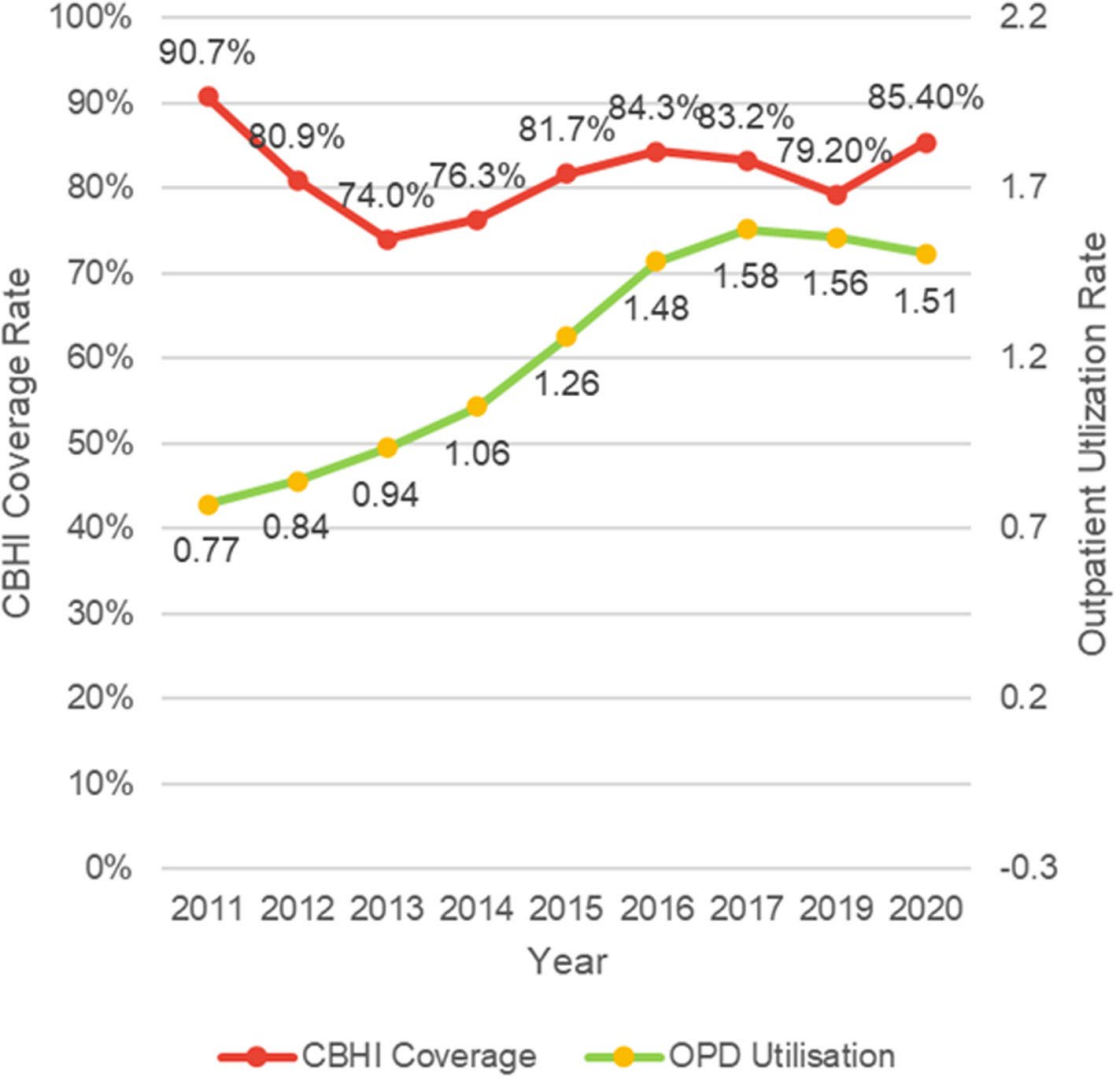
(**a**) Line graph comparing CBHI coverage and per capita OPD utilization rates in Rwanda. 2011–2020 (**b**) CBHI coverage rate, the red line, shows how the percentage of the population eligible for community-based health insurance varied over the years (the denominator excludes population already covered by private and other government insurance schemes). The early decline in 2013 corresponds with shift of the program from decentralized facility-level management to centralized management by the Ministry of Health. The second decline beginning in 2016 corresponds to the introduction of a 3-tiered premium structure based on income classification and transition of the CBHI scheme to the Rwanda Social Security Board (RSSB). The slight increase in 2020 corresponds to the widespread roll-out of the 3MS system. (**c**) The green line represents the more or less steady increase in outpatient care utilization rates from hospitals and health centers as new facilities were opened and access to care facilitated by simplifying CBHI enrollment/reenrollment processes

Rwanda has seen significant increases in service utilization rates at health facilities over the past decade, but they do not appear to correlate well with temporal trends in CBHI coverage (see Fig. 4). There were potentially other confounding interventions in the health sector during the same period that may have impacted service utilization rates. For example, digital tools helped to reduce perceived opportunity cost in traveling to the health facility while they also improved patient flow and wait times. Other interventions such as the establishment of new public and private health facilities, the national hospital accreditation program, improved supply chains for essential medicines/health commodities, and wide-ranging capacity-building initiatives for health workers have also contributed to increases in access to and quality of care that may have also driven up utilization rates.

In Kenya, several respondents from i-PUSH facilities reported rapid increases in the number of clients accessing their services. The clients seemed to be seeking comprehensive health care services in situations where they might otherwise only visit a pharmacist previously. Likewise, the majority of beneficiaries of the i-PUSH program who participated in the study had no access to any form of health insurance coverage prior to the program and affirmed that their service utilization had increased once they were enrolled.

## Discussion

### Research summary

These programmatic case studies provide rich qualitative information about a broad range of implementation challenges faced when implementing DFS programs for health at scale in LMIC contexts. They also describe how key challenges were mitigated and highlight programmatic results.

The case studies reinforced key conclusions found in the literature^3^, especially those related to enabling factors, such as the level of maturity of enterprise architecture (interoperability) and overall digital/ICT ecosystems; the importance of political mandates that expedite a hospitable regulatory environment; the role of digital platforms to facilitate participation in national health insurance; and the role of mobile money accounts to help people smooth health and non-health expenditures when faced with a health shock.

Our literature review identified multiple studies related to the expansion of the Rwandan CBHI program and its impact on UHC^12^, but none focused on specific technological innovations such as the introduction of DFS. In contrast, the majority of the Kenya studies did focus on technical DFS innovations^13^, yet none of them focused specifically on using the technology to promote progress toward UHC. These case studies help to fill some of the gaps.

### Recommendations

Five main recommendations grew out of the study findings:

- Use a whole systems approach to assess and build upon the existing digital landscape and engage stakeholders to build trust, align interests and enable data sharing that is needed for systems interoperability
- Directly address issues related to data security and privacy to facilitate data sharing and trust through the adoption of comprehensive health data security guidelines and health worker capacity building on managing protected health information (PHI).
- Promote opportunities to responsibly use the abundance of detailed transactional data generated by DFS for other purposes such as expanding access to other social protection schemes or improving health facility credit worthiness
- Use DFS to help expand financial protection based on the financial realities of the target populations served, enabling users to spread out payments over time and connect low-income households to social benefit schemes that can subsidize insurance premiums
- Consider incorporating DFS into healthcare financing components of resilience initiatives, as demonstrated by COVID-19 initiatives

### Limitations & further research

As a study limitation, the qualitative methodology used for this study was appropriate for the study questions but did not enable the attribution of benefits perceived to DFS technological innovations themselves. There were many simultaneous reforms taking place within the two health systems that were just as likely to have played a role in expanding UHC coverage and health service utilization. Another limitation was that most of the key informants were directly involved with program implementation so they may have been subjective in the benefits they perceived. (Our attempts to interview individuals who dropped out of the i-PUSH and MCF programs in Kenya were not successful when they refused to participate, apparently over concern that we would try to recover money from their unpaid premiums or loan reimbursements).

## Conclusion

The programmatic case studies of DFS for health programs described in this manuscript enable us to better understand the rationale for specific DFS interventions, highlight implementation issues encountered and draw out lessons learned and recommendations to inform future DFS initiatives to support progress toward universal health coverage.

They have also highlighted the wide range of benefits that can accrue to the general population, health service providers and public and private sector organizations that support the DFS ecosystem when health programs collaborate to incorporate digital financial services into their health interventions. These case studies supported some of the conclusions from the LHSS systematic review^2^ that advances in digital technology have made it more efficient and affordable to reach people with key health services by smoothing out health and non-health expenditures, yielding operational and cost efficiencies for provider payments as well as insurance enrollment and verification, and contributing to improvements in service quality. The findings and recommendations are particularly relevant at this time as many LMICs are seeing the confluence of two trends: a dramatic increase in mobile phone penetration and governments increasingly keen on expanding and digitizing health financing mechanisms to promote UHC, such as community-based health insurance.

## Data Availability

The datasets generated and/or analyzed during the current study are publicly available on USAID’s Data for Development Library, and from the corresponding author on reasonable request.

## Abbreviations

3MS: Mutuelle Membership Management System
API: Application program interface
CA: Cash Advance – unsecured mobile loan program funded by MCF
CBHI: Community Based Health Insurance
CCT: Conditional cash transfer
CHV: Community health volunteers
CHW: Community health worker
DFS: Digital financial service(s)
EMR: Electronic medical record
FINTECH: Companies that support the nexus between financing and technology
HMIS: Health management information system
HAS: Health savings account
ICT: Information and communication technology
IFHA: Investment Funds for Health in Africa
IHSSP: Rwanda Integrated Health Systems Strengthening Project
i-PUSH: Innovative Partnership for Universal Sustainable Healthcare
Irembo: Rwandan ‘gateway’ for government digital financial services
KIIs: Key informant interviews
LHSS: Local Health System Sustainability Project
LMIC: Low- and middle-income countries
LODA: Local Administrative Entities Development Authority (Rwanda)
MAF: Mobile Asset Finance – secured mobile loans for equipment purchases funded by MCF
MCF: Medical Credit Fund
MNO: Mobile network operator
MOH: Ministry of Health
M-PESA: Mobile phone-based money transfer service, mobile ‘cash’ (Kiswahili),
MSH: Management Sciences for Health
M-TIBA: Mobile ‘care’ (Kiswahili)
NHIA: National Health Insurance Authority
NHIF: National Hospital Insurance Fund (Kenya)
NIDA: National Identification Agency, Rwanda
OOP: Out-of-pocket
PHI: Protected Health Information
RHSS: Rwanda Health Systems Strengthening Activity
RSSB: Rwanda Social Security Board
SaaS: Software as a service
SACCO: Savings and credit cooperative
SME: Small and medium‐size enterprises
SMS: Short messaging service
UHC: Universal health coverage
USAID: United States Agency for International Development
USSD: Unstructured supplementary service data
WRA: Women of reproductive age

## Footnotes

1 World Health Organization: Universal Health Coverage. See citation [1] in references.

2 Mangone E, Riley P, and Datari K. 2021. See citation [2] in references.

3 World Health Organization: Sustainable Development Goals. See citation [3] in references.

4 As stated in Mangone E, Riley P, and Datari K. (2021, pg. iv.), the rapid proliferation of mobile telephones and advances in digital financial technology have created opportunities to accelerate progress toward UHC by expanding financial inclusion in LMICs.  See citation [2] in references.

5 Source World Bank DataBank (https://data.worldbank.org/indicator/IT.CEL.SETS, International Telecommunication Union ( ITU) World Telecommunication/ICT Indicators Database) 5/25/2021.

6 Some health facility managers in Kenya were interviewed both as implementers for the insurance program and as beneficiaries of the digital loan scheme (MCF).

7 Tellez, Z., & Zetterli, P. 2014. See citation [4] in references.

8 m-TIBA is a digital platform for inclusive healthcare that directly connects patients, providers, and payers such as family members, health insurers or donor agencies. It enables people to save, send, receive, and pay money for medical treatment through a mobile health wallet on their phone in a closed loop with conditional funds that can only be spent on healthcare at selected providers. Since it was introduced in Nairobi in 2019, more than 300,000 people have already signed up.

9 Gale NK, Heath G, Cameron E, Rashid S, Redwood, S. 2013. See citation [5] in references.

10 The name Irembo comes from the Kinyarwanda term for ‘gateway’ and is the government portal for digital financial services.

11 The name Ubudehe comes from the traditional Rwandan practice and cultural value of working together to solve problems. It refers in this case to a national income classification database managed by Rwanda’s Local Organizations Development Authority.

12 See citations [2, 5–13] in References.

13 See citations [14–20] in References.
